# Draft Whole-Genome Sequences of 16 Campylobacter jejuni Isolates Obtained from Wild Birds

**DOI:** 10.1128/MRA.00359-19

**Published:** 2019-06-27

**Authors:** Lisa Carraro, Francesca Marotta, Anna Janowicz, Claudio Patavino, Alessandra Piccirillo

**Affiliations:** a Department of Comparative Biomedicine and Food Science, University of Padua, Padua, Italy; b National Reference Laboratory for *Campylobacter*, Istituto Zooprofilattico Sperimentale dell’Abruzzo e del Molise Giuseppe Caporale, Teramo, Italy; University of Arizona

## Abstract

The draft genome sequences of 16 Campylobacter jejuni isolates obtained from wild birds are presented in this study. These genomes provide insights into the genetic features of C. jejuni isolates from wild birds, which are considered common hosts of this microorganism but have scarcely been investigated to date.

## ANNOUNCEMENT

Campylobacter jejuni is a leading foodborne pathogen worldwide (1). In the European Union, Campylobacter spp. account for over 200,000 human campylobacteriosis cases annually (2). In addition to being an important human pathogen, C. jejuni is frequently isolated from domestic and wild mammals and birds (3). Wild birds are recognized as common carriers of *Campylobacter* spp. and may play a role in their zoonotic transmission (4–7). However, the extent of their contribution to *Campylobacter* epidemiology is still largely unknown. Molecular typing is a powerful tool for studying *Campylobacter* epidemiology. Given its ability to generate high-throughput data, whole-genome sequencing (WGS) provides in-depth knowledge about genetic diversity and host adaptation of *Campylobacter* spp. (8).

Here, we report the draft whole-genome sequences of 16 C. jejuni isolates obtained from cloacal swabs of healthy wild birds in northern Italy between 2011 and 2016 (Table 1). The isolation and identification of *Campylobacter* spp. was performed as previously described (9). Genomic DNA (gDNA) was extracted from pure cultures (obtained from single colonies) using the Invisorb Spin tissue minikit (Stratec Molecular GmbH, Birkenfeld, Germany), and gDNA libraries were prepared using the Nextera XT library prep kit (Illumina, Inc., San Diego, CA). gDNA libraries were then sequenced using a NextSeq 500 sequencer (Illumina), with a read length of 150-bp paired-end reads.

**TABLE 1:** Genome characteristics and accession numbers of C. jejuni isolates obtained from wild birds

Isolate ID by order^*a*^	Yr of isolation	Host		No. of raw reads	% Q30 trimmed reads	Genome coverage (×)	*N*_50_ after scaffolding (bp)	Estimated genome length (bp)	G+C content (%)	No. of contigs by RAST	Largest contig size (bp)	No. of CDSs by RAST	No. of subsys tems by RAST	MLST profile	SRA accession no.	GenBank accession no.
		Common name	Scientific name													
Passeriformes																
US25	2011	Hooded crow	*Corvus cornix*	6,052,226	97.78	219	117,102	1,861,257	30.1	179	408,206	2,077	199	9732^*b*^	SRR9077768	RYYM00000000
US54	2012	Hooded crow	*Corvus cornix*	2,301,508	96.24	150	145,571	1,649,404	30.5	125	224,733	1,819	205	42	SRR9077776	RYYG00000000
CH246	2015	Hooded crow	*Corvus cornix*	1,203,250	96.35	79	157,739	1,617,704	30.4	44	304,071	1,720	199	4755	SRR9077765	RYYA00000000
US18	2011	Common blackbird	*Turdus merula*	2,136,040	97.85	82	61,187	1,912,650	30.1	451	183,560	2,505	203	9747^*b*^	SRR9077770	RYYO00000000
US55	2012	Common blackbird	*Turdus merula*	5,886,446	96.47	337	152,666	1,588,157	30.4	36	315,873	1,633	200	2538	SRR9077763	RYYF00000000
US50	2012	Western jackdaw	*Coloeus monedula*	5,026,502	96.35	336	132,116	1,830,203	30.0	128	266,764	2,009	208	9746^*b*^	SRR9077771	RYYJ00000000
US51	2012	Western jackdaw	*Coloeus monedula*	7,970,236	96.36	493	184,120	1,599,320	30.5	35	554,142	1,633	205	267	SRR9077772	RYYI00000000
CH186	2015	Eurasian jay	*Garrulus glandarius*	3,920,140	96.29	255	165,915	1,596,428	30.4	34	554,125	1,632	200	2538	SRR9077778	RYYB00000000
US53	2012	Carrion crow	*Corvus corone*	9,048,224	96.23	596	146,127	1,588,911	30.4	49	419,775	1,634	200	177	SRR9077775	RYYH00000000
CH182	2015	Eurasian magpie	*Pica pica*	5,201,042	96.13	356	211,630	1,654,792	30.5	50	390,030	1,728	209	45	SRR9077777	RYYD00000000
Strigiformes																
US12	2011	Little owl	*Athene noctua*	1,028,246	97.46	37	152,970	1,703,137	30.3	52	226,920	1,805	203	45	SRR9077769	RYYP00000000
US24	2011	Tawny owl	*Strix aluco*	4,800,604	97.67	228	207,971	1,677,087	30.5	48	339,079	1,783	202	220	SRR9077767	RYYN00000000
Gruiformes																
US33	2011	Water rail	*Rallus aquaticus*	3,650,720	96.22	245	180,616	1,923,707	30.7	62	232,159	2,017	199	ND^*c*^	SRR9077773	RYYL00000000
Charadriiformes																
US42	2012	Yellow-legged gull	*Larus michahellis*	1,474,096	96.12	100	221,533	1,631,410	30.5	29	419,087	1,670	202	2353	SRR9077774	RYYK00000000
Apodiformes																
CH165	2015	Common swift	*Apus apus*	5,061,820	96.36	322	210,269	1,603,498	30.5	37	357,107	1,654	204	9478	SRR9077764	RYYE00000000
Columbiformes																
CH278	2016	Rock dove	*Columba livia*	4,495,308	96.39	292	174,046	1,645,481	30.4	55	339,321	1,738	202	2209	SRR9077766	RYXZ00000000

a ID, identifier.

b New ST (the US18 isolate was also assigned to new alleles, *tkt* 773 and *glnA* 706).

c ND, not determined.

Raw reads were *de novo* assembled using SPAdes 3.11.1 (settings, k-mer sizes 21, 33, 55, and 77 with mismatch careful mode) (10), and contigs of <200 bp were discarded using Geneious Prime 2019.0.4 (Biomatters ApS, Aarhus, Denmark). The QUAST software (11) was used to evaluate genome assembly quality. Annotation of the genomes was performed using the NCBI Prokaryotic Genome Annotation Pipeline (PGAP) (12) and the Rapid Annotations using Subsystems Technology (RAST) server (13). The genomes were also analyzed using MLST 2.0 to identify multilocus sequence typing (MLST) profiles (14). ResFinder 3.1 was used to identify resistance genes (15), and PlasmidFinder 2.0 was used to search for plasmids (16). BacWGSTdb (17) was also used to predict the presence of virulence and resistance genes. Default parameters were used for all software unless otherwise specified.

The 16 draft genomes were assembled into 29 to 451 contigs, with accumulated lengths ranging from 1.58 to 1.92 Mbp and an average G+C content of 30.4% (Table 1). The annotated genomes by RAST revealed 199 to 209 subsystems, 1,632 to 2,505 coding sequences (CDSs), and 42 to 45 RNAs. About half of the isolate genomes contained clustered regularly interspaced palindromic repeat (CRISPR) systems, but no plasmids were identified. MLST analysis assigned isolates to three novel sequence types (STs) and 12 STs previously identified in C. jejuni from humans, animals (including wild birds), and the environment (https://pubmlst.org/campylobacter/). Several genes associated with virulence (e.g., *fliA*, *fliF*, *fliK*, *fliM*, *fliY*, *flgE*, *flgH*, *flgI*, and *rpoN* genes for motility; *cheA*, *cheV*, *cheW*, and *cheY* genes for chemotaxis; *cadF*, *jlpA*, *pebA*, and *flpA* for adhesion; *flhA*, *flhB*, *fliP*, *fliQ*, *fliR*, *flaC*, *ciaB*, and *ciaC* for invasion; *cdtA*, *cdtB*, and *cdtC* for toxin production; *pgl* for glycosylation; and *chuA* for iron uptake) (18) were identified in most isolates that also carried genes, alone or in combination, encoding β-lactam (*bla*_OXA-61_, *bla*_OXA-185_, *bla*_OXA-446_, *bla*_OXA-447_, *bla*_OXA-448_, and *bla*_OXA-449_), tetracycline [*tet*(O)], and aminoglycoside [*aph*(*3′*)*-III*] resistance. The presence of virulence and resistance genes in C. jejuni isolates from wild birds are concerning and need to be further investigated.

The draft whole-genome sequences of the 16 C. jejuni isolates reported in this study are the first from wild birds in Italy. They will help in understanding the molecular epidemiology of C. jejuni in wild bird populations.

### Data availability.

The draft whole-genome sequences and annotations are publicly available at the NCBI GenBank database under the accession numbers presented in Table 1. Raw reads can be found under the NCBI SRA BioProject number PRJNA510785.
